# Advances in nanocarriers to improve the stability of dsRNA in the environment

**DOI:** 10.3389/fbioe.2022.974646

**Published:** 2022-08-16

**Authors:** Wenling Yang, Baitao Wang, Gao Lei, Guocan Chen, Dehai Liu

**Affiliations:** Key Laboratory of Microbial Engineering at the Institute of Biology, Institute of Biology Co., Ltd., Henan Academy of Sciences, Zhengzhou, China

**Keywords:** RNA biopesticide, dsRNA, stability, nanomaterial, pest control

## Abstract

RNAi technology, known as a revolutionary technology in the history of pesticides, has been identified as a very promising novel approach for crop protection, which is of great significance for achieving the sustainable agricultural development of the United Nations Food and Agriculture Organization. Although many studies have shown that RNA biopesticides have strong application prospects, its stability seriously restricts the commercial use. As the core component of RNAi, double-stranded RNA (dsRNA) is unstable in its natural form. Therefore, how to ensure the stability of dsRNA is one of the most significant challenges in realizing the commercial use of RNA biopesticides. Nanomaterials such as cationic polymers and lipofectamine can improve the stability of dsRNA in the environment, which has been proved. This paper reviews the recent research progress of nanomaterials that can be used to improve the environmental stability of dsRNA, and discusses the advantages and limitations of different nanomaterials combined with dsRNA, which provides reference for the selection of dsRNA nanoformulations.

## 1 Introduction

Agricultural insect pests are one of the main threats that restrict stable agricultural production (Douglas, 2018). Since the discovery of dichlorodiphenyltrichloroethane (DDT) in 1939, synthetic pesticides have played a vital role in mainstream commercial farming around the world (Rezende-Teixeira et al., 2022). In recent years, with the increase in the frequency and time of the use of chemical pesticides, such problems as pesticide resistance and the re-emergence of insect pests have intensified, resulting in a decrease in the efficiency of insect pest control (Abbasi et al., 2022). Simultaneously, chemical pesticides have introduced many negative effects, including damaging soil and water resources (Hüesker and Lepenies, 2022; Rasool et al., 2022), harming human health and that of some non-target organisms, and decimating biodiversity (Wagner et al., 2021). Moreover, regulators in many countries and regions around the world are gradually restricting or banning the use of some chemical pesticides. For example, the European Commission has declared that it will reduce the total use of chemical pesticides by 50% by 2030 (Tataridas et al., 2022). Therefore, it is necessary to find more green, effective, eco-friendly and safe pesticides for crop protection.

RNAi technology is an effective strategy to control harmful microorganisms, insect pests, mites and nematodes, that reduces the use of pesticides and helps to achieve the goals of sustainable agricultural development (Pan and Zhang, 2020; Mosa and Youssef, 2021; Joshi et al., 2022). RNAi is a phenomenon of gene silencing that is mediated by double-stranded RNA (dsRNA), which can inhibit the expression of the target genes by hindering the translation or transcription of specific genes in insects, and finally achieve the purpose of killing the target pests (Velez and Fishilevich, 2018; Liu et al., 2020). dsRNA-based pesticides primarily act on target species in two manners in the field: host-induced gene silencing (HIGS) (Dalakouras and Ganopoulos, 2021; Hendrix et al., 2021) and spray-induced gene silencing (SIGS) (Saxena et al., 2022). In 2017, Monsanto developed the first RNAi-based crop against the western corn rootworm (*Diabrotica virgifera*) in the western and northern United States. This product is the insect-resistant genetically modified corn SmartStax-PRO, which has been approved by the United States Environmental Protection Agency (EPA) to control *D. v. virgifera* (Shaffer, 2020). The RNAi technology has not yet had a mature product on the market, and a stable delivery system is the core problem that restricts this technology. In this review, we will focus on advances in the stability of dsRNA as they transition from laboratory to field environments and discuss successes in improving the environmental stability of dsRNA.

## 2 Obstacles to the commercialization of dsRNA-based biopesticides

The environmental stability of dsRNA is a key factor that affects the application of RNA biopesticides (Martinez et al., 2021). Therefore, the transition of dsRNA from the laboratory to field environment needs to be optimized to improve its stability in the environment. The field environment is more complex and constantly changing, and the dsRNA is degraded or taken up by multiple pathways before being ingested by insect pests (Bachman et al., 2020; Qiao et al., 2021). For example, dsRNA alone can be completely degraded in soil and water environments within 48 h. Environmental factors such as nucleases, rainwater, ultraviolet rays, and microorganisms directly affect the stability of dsRNA (Rank and Koch, 2021). The stability of dsRNA will face threats from the insect body after it has been ingested. This stability varies among insect species. The pH in insect gut has a substantial influence on the efficiency of RNAi (Cooper et al., 2020), and the pH in midgut is known to vary greatly among insects (Cooper et al., 2019). For example, the guts of Coleoptera and Hemiptera are weakly acidic, while those of Orthoptera, Diptera, and Hymenoptera are alkaline. The stability of dsRNA is greatly affected by the pH, and it is generally stable at pH 4.0–5.0 (Romeis and Widmer, 2020). Therefore, dsRNA is more stable in the guts of Coleoptera and Hemiptera after ingestion (Wynant et al., 2014) but less stable in those of Orthoptera, Diptera, and Hymenoptera (Cooper et al., 2020). In addition, dsRNA can be degraded by nucleases in the insect gut and lymph or by commensal microorganisms (Christiaens et al., 2014; Wynant et al., 2014), resulting in low efficiency of RNAi. Nucleases are the primary factor that affects the stability of dsRNA in insects. The gut, lymph, and whole body of insects can secrete nucleases, and the enzymatic activity in gut tissue is significantly higher than that in other parts, such as the lymph and entire body. The stability of dsRNA varies with the differential nuclease activities of insects. The nuclease activity in the lymph of lepidopteran insects, such as *Spodoptera litura* is higher, while the activity of nuclease in the gut of Orthoptera, such as *Locusta migratoria* was higher. Therefore, dsRNA is less stable in Lepidoptera (*S. litura*) when the dsRNA is delivered by injection. However, dsRNA in the gut of Orthoptera (*L. migratoria*) is unstable when it is delivered by oral ingestion (Peng et al., 2018). Moreover, the obstacles to the commercialization of RNA pesticides also include a high cost of production and low efficiency of delivery.

## 3 Nanocarriers -- a viable solution for dsRNA stabilization

**FIGURE 1:**
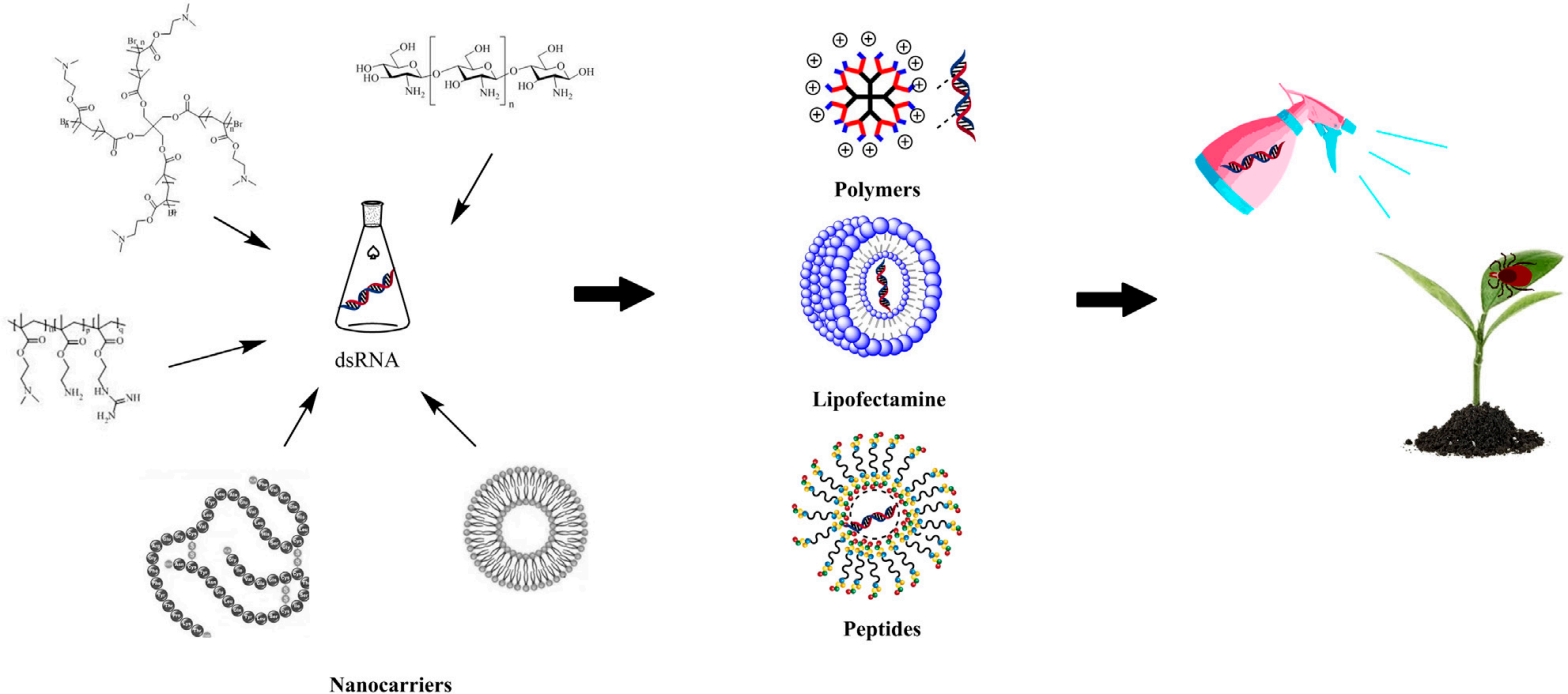
Application of nanocarriers in improving the stability of dsRNA.

Nanocarriers can greatly enhance the efficiency of delivery of pesticides, improve the bioactivity of synthetic pesticides and decrease the amount of pesticide residues (Jiang et al., 2022; Yan et al., 2022), thus, showing great potential for green pest management (Dong et al., 2022). Shen jie’s team conducted research on the mechanism and delivery process of RNAi mediated by nanoparticles and proposed that the efficient protection and delivery of dsRNA by nanocarriers was the key factor that enhanced the efficiency of RNAi (Ma et al., 2022). The formulation of nanocarriers with dsRNA to build a stable and efficient dsRNA delivery system produced a solution for the stable existence of dsRNA in the environment (Barros et al., 2019; Linyu et al., 2021) (Figure 1). Nanocarriers has several significant advantages. First, the complexation of dsRNA and nanocarriers can isolate the dsRNA from an unfavorable external environment, overcome the adverse effects of ultraviolet radiation, destruction by rain, nuclease degradation and other factors (Parker et al., 2019) and improve the stability and adhesion of dsRNA (Kolge et al., 2021). Second, owing to the mechanism by which dsRNA targets specific gene sequences, it can reduce the risk to non-target organisms. The targeting of dsRNA can be improved by a coating of nanocarriers. When the nanoformulation is sprayed on foliage, irrigated to the roots of the crops or used to treat seeds, it will also be highly selective (Bramlett et al., 2020) and can release the dsRNA to specific targets (Rank and Koch, 2021). Third, the use of nanomaterials as dsRNA delivery carriers helps to increase the absorption and uptake of insect cells, which aids the ability of exogenous dsRNA to penetrate barriers such as the peritrophic membrane and body wall of the gut of insect pests, and improve its insecticidal efficiency (Saxena et al., 2022). Fourth, nanocarriers can also improve the sensitivity of dsRNA that are sprayed on crops, which significantly improves the sensitivity toward and effect on the target insects (Das et al., 2015; Cooper et al., 2019; Martinez et al., 2021).

## 4 Nanocarriers that improve dsRNA stability

Nanocarriers currently used for dsRNA primarily include polymers, lipofectamine, peptides and other compounds (Table 1).

**TABLE 1:** Various nanocarriers to improve the stability of dsRNA.

Target insects	Nanocarriers	Target genes	Improve stability	Improve RNAi efficiency	References
*Helicoverpa armigera*	Chitosan	*juvenile hormone methyltransferase (JHAMT) and acetylcholine esterase (ACHE)*	Yes	Yes	Kolge et al. (2021)
*Anopheles gambiae*	Chitosan	*chitin synthase* genes, *AgCHS1* and *AgCHS2*	-	Yes	Zhang et al. (2010)
*Aedes aegypti*	Chitosan	*wing development vestigial (vg) gene*	Yes	Yes	Ramesh Kumar et al. (2016)
*Aphis gossypii*	star polycation	*CYP6CY3*	Yes	Yes	Linyu et al. (2021)
*Chilo supperssalis*	star polycation	*CYP15C1*	Yes	Yes	Sun et al. (2020)
*Aphis glycines*	star polycation	*TREH*, A*TPD*, *ATPE* and *CHS1*	-	Yes	Yan et al. (2019)
*Myzus persicae*	star polycation	*vestigial* (*vg*) and *Ultrabithorax* (*Ubx*)	-	Yes	Zhang et al. (2021)
*Spodoptera exigua*	guanidine-containing polymers	*chitin synthase B* gene (*CHSB*)	Yes	Yes	Christiaens et al. (2018)
*Aedes aegypti*	sodium tripolyphosphate cross-linked chitosan complexes	*IAP1*, *SNF7*, *SRC*, *SSK*, MESH, *HEL25E*, *SAC1*, *LRC, OTK*	Yes	Yes	Dhandapani et al. (2019)
*Chilo supperssalis*	chitosan、lipofectamine 2000, carbon quantum dots	glyceraldehyde-3-phosphate dehydrogenase gene (*G3PDH*)	Yes	Yes	Wang et al. (2020)
*Euschistus heros*	Liposome	*vATPase A* and *muscle actin*	Yes	Yes	Castellanos et al. (2019)
*Blattella germanica*	Liposome	*α-tubulin*	Yes	Yes	Huang et al. (2018)
*Spodoptera frugiperda*	Cellfectin II	*IAP*	Yes	Yes	Gurusamy et al. (2020)
*Anthonomus grandis*	fusion peptide	*A. grandis chitin synthase II* gene (*Ag-ChSII*)	Yes	Yes	Gillet et al. (2017)
*Aedes aegypti*	carbon quantum dots	*SNF7* and *SRC*	Yes	Yes	Das and Sherif, (2020)
*Apis mellifera*	cationic perfluorocarbon (PFC) nanoparticles	*DNA methyltransferase 3* (*dnmt3*) gene	Yes	Yes	Li-Byarlay et al. (2013)

### 4.1 Macromolecular polymer

Macromolecular polymers disperse very effectivity and are stable, and the complexation of dsRNA with macromolecular polymers can increase the stability and targeting efficiency of dsRNA. The different molecular structures of polymers enable them to be divided into the categories of chitosan, core-shell nanocarriers, star polycation and guanidine-containing polymers.

#### 4.1.1 Chitosan

Chitosan is a macromolecular polymer that is similar in structure to cellulose. Chitosan can connect with negatively charged dsRNA through electrostatic forces owing to the positive charge on its surface to form nanoparticle complexes (Ramesh Kumar et al., 2016). dsRNA mediated by chitosan nanoparticles can resist degradation by nucleases and the insect gut and improve the stability of dsRNA in the gut and its efficiency at entering the lymph (Kolge et al., 2021). This primarily ensures the stability of dsRNA in the gut of *Spodoptera frugiperda* and *Ostrinia nubilalis* and improves the silencing efficiency of insect target genes (Wang et al., 2020; Cooper et al., 2021). A recent study showed that chitosan derivatives cross-linked with sodium tripolyphosphate can be used to enhance the efficiency of RNA interference against *Aedes aegypti* (Dhandapani et al., 2019), and the mortality rate can reach more than 70%. In addition, chitosan and its derivatives are advantageous owing to their inexpensive production, biodegradability and generally environmental friendliness, while chitosan will combine with some negatively charged non-specific proteins, which require the structural modification of this nanomaterial.

#### 4.1.2 Core-shell nanocarriers

Core-shell nanocarriers are nanoscale ordered structures formed by one type of nanomaterial that encapsulates another type of nanomaterial. The structure is relatively stable and is widely used to deliver RNAi. For example, the efficiency of RNAi can be significantly improved by complexing cationic core-shell fluorescent nanoparticle with dsRNA to target the midgut-specific chitinase gene *CHT10* of the *Ostrinia furnacalis*. After 5 days of feeding, the normal development of *O. furnacalis* larvae was obviously hindered (He et al., 2013). In addition, Zheng et al. (2019) prepared a cationic core-shell fluorescent dendrimer that can deliver the dsRNA of the target gene into the cuticle of *Aphis glycines*, thereby improving the efficiency of gene silencing and control effect. Core-shell nanocarriers have more superior properties than single nanomaterials, but the high production cost of cationic core-shell fluorescent nanoparticles limits their potential application in the market.

#### 4.1.3 Star polycation

The nanocarrier star polycation (SPc) is a cationic dendrimer that consists of four peripheral amino acid functionalized arms, and the dendrimer can condense random nucleic acids into complexes that are easily taken up through endocytosis (Li et al., 2019; Zheng et al., 2019; Yan et al., 2019). Yan et al. (2019) replaced the fluorescent core to develop an inexpensive nanocarrier (SPc) to decrease its cost. Spraying a formulation of dsRNA/SPc/detergent increased the ability of dsRNA to penetrate the body wall of aphids by more than 3-fold with the help of nanocarrier, and the dsRNA carried by the nanoparticles successfully silenced the expression of target genes, ultimately causing up to 80% of mortality of the aphids. In addition, Sun et al. (2020) used a dsRNA mixture with SPc to target the cytochrome *P450 monooxygenase* gene *CYP15C1*, which can significantly increase the mortality of *Chilo supperssalis* larvae (Sun et al., 2020). Li et al. (2022) constructed an SPc-based gene/drug delivery system for co-delivering hemocytin (hem) dsRNA and botanical pesticide matrine to develop a novel multicomponent nano-pesticide with sequential bioactivity against devastating *Myzus persicae*. The multicomponent nano-pesticide successfully overcame the problems of short duration of RNA pesticides and slow effect of plant-based pesticides, and the ability to control this insect in the field increased by more than 90%. This shows that star-shaped cationic polymers can increase the stability of dsRNA and affect the retention time of dsRNA activity in the environment (Whitfield et al., 2018). Compared with dendrimers, SPc are simpler to synthesize, and their multi-arm structures are more easily modified. Currently, the cost of raw materials to construct SPc has been reduced further, and it has better prospects for application in the market (Li et al., 2019).

#### 4.1.4 Guanidine-containing polymers

Guanidine polymers can form stable complexes with dsRNA to improve its stability under strong alkaline conditions. Since the gut of some insects in the field environment is strongly alkaline, which greatly hinders the stability of dsRNA, guanidine polymer nanoparticles are usually used to overcome this unfavorable condition. For example, Christiaens et al. (2018) used DMAEMA and AEMA as building blocks to synthesize chain structures, and then used pyrazole-1-carboxamidine to modify a part of primary amine groups into guanidine groups. The guanidino-structured nanoparticles were applied to *Spodoptera exigua*, which significantly improved the stability of dsRNA and the efficiency of RNAi under strongly alkaline intestinal conditions.

### 4.2 Lipofectamine

Lipofectamine is a phospholipid bilayer with exposed cations, which can adsorb and coat dsRNA, and the outer cations can help to overcome the electrostatic repulsion between nucleic acid molecules and cell membranes, and this structure is very effective at promoting its binding to anions on the cell surface and then delivering dsRNA into the cell. The use of lipofectamine as a nanocarrier enables it to encapsulate nucleic acids into cells (Christiaens et al., 2020), and studies have shown that the presence of lipofectamine can improve the stability of dsRNA when treated with endonuclease (Gurusamy et al., 2020). Christiaens et al. (2020) studied the effect of applying lipofectamine as carriers on a variety of insects, including Diptera (*A. aegypti*), Hemiptera (*Euschistus heros*), Lepidoptera (*S. frugiperda*), and Blattaria (*Blattella germanica*), and the results showed a significant improvement in the stability and efficiency of dsRNA. Gurusamy et al. (2020) used Cellfectin II combined with dsRNA to target the inhibition of apoptosis protein 1 expression in Drosophila. Cellfectin II increased the stability of dsRNA, which resulted in the repression of larval target genes and increased mortality after feeding. In addition, by silencing the *G3PDH* gene in *C. supperssalis*, lipofectamine 2000 improved the stability and cellular uptake of dsRNA, resulting in higher lethality in this insect (Wang et al., 2020). Therefore, lipofectamine is strongly biocompatible as a nanocarrier, which enhances delivery into insect cells, and is strongly applicable. Moreover, lipofectamine is non-toxic, easily prepared, and highly amenable to large-scale production. However, there are also some types of lipofectamine that have low transfection efficiency and are not easily absorbed by cells.

### 4.3 Peptide

The use of peptides as nanocarriers can enhance the RNAi efficiency of pests and improve the stability of dsRNA (Choi and David, 2014). One of the representative categories of peptide carriers is cell-penetrating peptides, which are short-chain cationic peptides that are composed of 10–30 amino acids that are primarily basic residues (Durzynska et al., 2015). dsRNA binds to the binding domain of cell-penetrating peptides to form ribonucleoprotein particles, which enter the cells through endocytosis and improve the efficiency of entering cells (Choi and David, 2014). Gillet et al. (2017) used the chimeric protein PTD-DRBD to combine with dsRNA to form a ribonucleoprotein particle, which improved the effectiveness of the RNAi mechanism of *Anthonomus grandis*. Compared with the direct use of dsRNA, it primarily limits the contact between dsRNA and nuclease and avoids the degradation of dsRNA by nuclease. Thus, improves the stability of dsRNA and its efficiency to enter cells, which results in a significant reduction in the transcription level of target genes. Recently, an amphiphilic polypeptide capsule, a nanocarrier for the self-assembly of polypeptides, was reported that is structurally stable and easily synthesized. The amphiphilic polypeptide capsules can promote the uptake of dsRNA by cells of the *Tribolium castaneum* and *Acyrthosiphon pisum*, and increase the mortality rate. Amphiphilic peptide capsules are similar to guanidine polymer nanoparticles in that they can also remain highly stable in strong alkaline environments (Barros et al., 2019). This nanocarrier is highly biocompatible and has strong abilities to target and penetrate cells, but its high production cost limits its marketability.

### 4.4 Other nanocarriers

Various other nanocarriers have also been used to improve the stability and efficiency of dsRNA in pest control. The type of nanocarrier needs to be adjusted for different target species to determine the most efficient combination of nanocarrier and dsRNA. In addition to chitosan and guanidine-containing polymers, carbon quantum dots (CQD) can also very efficiently protect dsRNA in alkaline environments, while complexes of amine functionalized silica nanoparticles (ASNP) and dsRNA are completely degraded under strong alkaline conditions (Das et al., 2015). In addition, the study found that the carrier loaded with dsRNA by coating includes not only lipofectamine but also other delivery vehicles. For example, anucleated minicells derived from *Escherichia coli* can be utilized as a cost-effective and scalable platform to produce and encapsulate dsRNA. The dsRNA (ME-dsRNA) encapsulated in a minicell was shielded from degradation by RNase and stabilized on strawberry surfaces, which allows dsRNA to persist in field-like conditions (Islam et al., 2021). AgroSpheres has developed new biological particles that are composed of small spherical cells that lack chromosomes, which can encapsulate dsRNA, protect dsRNA from environmental aggression, and be stably and continuously released. It can also make dsRNA firmly adhere to plant leaves to prevent rain erosion. The development of these nucleic acid-coated carriers has greatly facilitated the stability of dsRNA in the environment, thereby accelerating the commercialization of RNA biopesticides. However, considering that the development of plants in the field environment and those in the laboratory can differ, such as differential growth of leaf cuticles, the adhesion and stability of dsRNA on leaves could be affected (Elhaj Baddar et al., 2020).

## 5 Prospects

Although studies have shown that RNAi has great potential for plant protection, there are still many challenges in the promotion and application of RNA products in the market. For example, the rapid degradation of dsRNA has made it difficult to apply RNA biopesticides. Researchers have tried various strategies to improve its stability in the environment, and nanomaterials provide a new delivery platform for dsRNA-based biopesticides, and can significantly improve the efficiency of gene interference. Moreover, some nanomaterials are inherently toxic to some species, which can provide a new direction for the development of diversified and multi-faceted crop protection systems. Many experiments have confirmed the safety of nanomaterials at the cellular and *in vivo* levels (Xu et al., 2014; You et al., 2014; Gao et al., 2016). However, analyses of the safety of nanomaterials after environmental release still require further study (Kookana et al., 2014; Walker et al., 2019), particularly the potential cumulative toxicity of nanomaterials in the ecosystem, which subsequently penetrate the body of an organism in varying manners, causing potential threats. In conclusion, the pest control technology of nanotechnology combined with dsRNA has many natural advantages and can provide new strategies for pest control. However, the process of research and development still needs to consider many key issues.
